# Lessons for postgraduate trainees about Dhat syndrome

**DOI:** 10.4103/0019-5545.37324

**Published:** 2007

**Authors:** Om Prakash

**Affiliations:** Department of Psychiatry, National Institute of Mental Health and Neurosciences (NIMHANS), Bangalore - 560 029, Karnataka, India

**Keywords:** Clinical profile, Dhat syndrome, management

## Abstract

Dhat syndrome (“semen loss”-related psychological distress) is a culture-bound syndrome seen in the natives of Indian subcontinent, but it is prevalent in other cultures also. Its diagnosis and management issues need to be taught to postgraduates in their teaching program. This syndrome involves vague and multiple somatic and psychological complaints such as fatigue, listlessness, loss of appetite, lack of physical strength, poor concentration, forgetfulness and other vague somatic troubles. These symptoms are usually associated with an anxious and dysphoric mood state. These patients may also present with or without psychosexual dysfunction. The management of Dhat syndrome needs serious attention. The understanding of this condition by Modern Medicine fails to impress most of the patients, and the explanations and reassurances offered prove to be not of much use.

Our cultural perspective can shape the experience and understanding of mental illness. Because of these cultural influences, Yap[1] coined the term “culture-bound syndromes,” which seem to be episodic, dramatic and discrete patterns of behavioral reactions specific to a particular community that articulate both personal predicament and public concerns.[2]

## CONCEPT OF DHAT

The word “Dhat” derives from the Sanskrit language (the mother of Indo-Aryan languages) word *dhatu*, meaning “metal,” “elixir” or “constituent part of the body” which is considered to be “the most concentrated, perfect and powerful bodily substance, and its preservation guarantees health and longevity.” The disorder related to this *dhatu*, i.e., semen, is mentioned in ancient treatise “Susruta Samhita” as *shukrameha* (*shukra* = sperm; + *meha* = passage in urine).

Since then, myth prevalent among people of the Indian subcontinent is that “it takes 40 days for 40 drops of food to be converted to one drop of blood, 40 drops of blood to make one drop of bone marrow and 40 drops of bone marrow form one drop of semen.”[3]

It is important to learn that this anxiety around seminal loss is not only prevalent in the Indian subcontinent but also in the Western world. From the times of Hippocrates and Aristotle, semen is considered an extremely important part of the body. “Sperms are the excretion of our food; or to put it more clearly, the most perfected component of food” (Aristotle, 384-322 B.C.). Andrew Tissot (1728-97) commented in his treatise on the disease produced by onanism that “losing one ounce of sperm is more debilitating than losing 40 ounces of blood.” His statement seems to be closer to the Indian myth.

In many Western European cultures, masturbation was prohibited by religion. Henry Maudsley (1835-1918) even considered that semen loss, especially if it occurs through masturbation, results in serious mental illness. George Beard (1838-1883) considered nocturnal emissions of semen as one of the commonest reasons for neurasthenia. Therefore, the concept of Dhat syndrome or semen-loss syndrome was prevalent among Western cultures with different names at some point of time.

## DESCRIPTION OF DHAT SYNDROME

Wig[4] coined the term “Dhat syndrome,” characterized by vague somatic symptoms of fatigue, weakness, anxiety, loss of appetite and guilt attributed to semen loss through nocturnal emissions, urine and masturbation though there is no evidence of loss of semen. This notion of seminal loss frightens the individual into developing a sense of doom if a single drop of semen is lost, thereby producing a series of somatic symptoms.[5] Thus, in this syndrome, hypochondriacal, anxiety and depressive symptoms become subsumed in the major visible “pathology” of semen loss.[6] Currently this syndrome lies with DSM IV- Appendix I- culture-bound syndromes[7] and under “other specific neurotic disorders” (F48.8) in ICD-10.[8]

## EPIDEMIOLOGY

This syndrome is more prevalent in the Indian subcontinent[5,9–11]; therefore, it is considered as “neurosis of the orient” but it showed global presence, like “shen-k'uei” in China.[12,13] There are enough historical evidences that similar kinds of syndromes were prevalent in Europe, USA and Australia in the 19th century, which disappeared in response to changes in social and economic factors.[14,15]

## CLINICAL PROFILE

The patients who presented with symptoms of Dhat syndrome were mostly young, recently married, belonging to average or low socioeconomic status (perhaps a student, laborer or farmer by occupation), from rural area and from family with conservative attitudes towards sex.[12,16,17]

Patients having Dhat syndrome can be further divided into three categories.[18]

1. Dhat alone - Patients attributed their symptoms to semen loss; presenting symptoms - hypochondriacal, depressive or anxiety symptoms
2. Dhat with comorbid depression and anxiety - Dhat was seen as an accompanying symptom
3. Dhat with sexual dysfunction

The duration of presentation of these patients from the onset varies from less than three months up to one year,[5,16] even up to 20 years.[10] These patients reported that they lose their semen in sleep, with urine, masturbation, hetero/homosexual sex.[10,16,17,19]

Erectile dysfunction (22-62%) and premature ejaculation (22-44%) were the most commonly associated psychosexual dysfunctions; while depressive neurosis (40-42%), anxiety neurosis (21-38%), somatoform/hypochondriasis (32-40%) were the most reported psychiatric disorders in the patients having diagnosis of Dhat syndrome.[5,10,11,16,17,20,21]

Urine examination of these patients did not reveal any abnormality except oxaluria (10%) and phosphaturia (6%).[5] They also showed significant different illness-related beliefs and behaviors compared to controls and had similarities with other functional somatic syndromes.[11,22]

On follow-up of these patients, a majority of patients recovered (66%), while the rest either improved (22%) or were unchanged (12%).[16] Dissatisfaction with medical explanation can be extrapolated, as majority of them did not attend after the initial visit.[5,20]

## KNOWLEDGE AND ATTITUDE

Regarding composition, majority of patients believed that Dhat consisted of semen, followed by pus, sugar, concentrated urine, infection or “not sure.” Majority considered masturbation and/or excessive indulgence in sexual activities as important causative factor, followed by venereal diseases, urinary tract infections, overeating, constipation or worm infestation, disturbed sleep or genetic factors.[16,17]

Majority got the information about Dhat syndrome from friends, colleagues or relatives. Fear of semen loss and its cure are propagated by *vaids* and *hakims* and advertised everywhere on walls, on television, in newspapers and on roadside hoardings in most of the northern Indian cities. Most of the patients prefer to visit STD clinics, urologists and physicians rather than approach psychiatrists.

## MANAGEMENT

Understanding of Dhat syndrome by Modern Medicine fails to impress most patients. Wig[4] suggested emphatic listening, a nonconfrontational approach, reassurance and correction of erroneous beliefs, along with the use of placebo, anti-anxiety and antidepressant drugs, wherever required. Other group advocated psychoeducation and culturally informed cognitive behavioral therapy.[15] Good response was reported with anti-anxiety and antidepressant drugs as compared to psychotherapy.[17] Depressive symptoms of this syndrome showed effective response to selective serotonin reuptake inhibitors along with regular counseling.[21]

The available intervention studies suggest that the management of Dhat syndrome involves sex education, relaxation therapy and medications.[23]

Sex education primarily focuses on anatomy and physiology of sexual organs and their functioning with reference to masturbation, semen, nocturnal emissions. It also involves functioning with genitourinary system independent of gastrointestinal tract, etc. Relaxation therapy mainly consists of Jacobson's Progressive Muscular Relaxation Technique, which can be combined with biofeedback (so as to facilitate objective evidence and mastering of anxiety by the patient). Relaxation therapy should be practiced two to three times per day regularly, especially after therapy sessions are over.

Evaluation of the presence of associated anxiety or depressive symptoms that may impede the process of therapy must be performed. Anxiolytics or/and antidepressants can be added for the least possible time and in the least possible doses.

## CONCLUSION

Dhat as a symptom is important for assessment of psychosexual problems, but the classification of Dhat syndrome is problematic. Symptoms related to Dhat syndrome are also found in populations other than oriental populations. There is need for more research in this neglected area to find its right place in nosology.
